# Contribution of *Tomato torrado virus* Vp26 coat protein subunit to systemic necrosis induction and virus infectivity in *Solanum lycopersicum*

**DOI:** 10.1186/s12985-019-1117-9

**Published:** 2019-01-14

**Authors:** Przemysław Wieczorek, Barbara Wrzesińska, Patryk Frąckowiak, Arnika Przybylska, Aleksandra Obrępalska-Stęplowska

**Affiliations:** Department of Entomology, Animal Pests & Biotechnology, Institute of Plant Protection-National Research Institute, Władysława Węgorka 20 St, 60-318 Poznań, Poland

**Keywords:** Coat protein, *Torradovirus*, Pathogenicity determinant, Plant defense, *Solanum lycopersicum* L., Systemic necrosis, Plant-virus interaction

## Abstract

**Background:**

*Tomato torrado virus* (ToTV) infection manifests with burn-like symptoms on leaves, leaflets and upper stem parts of susceptible infected plants. The symptoms caused by ToTV may be considered as one of the most severe virus-induced forms of systemic necrosis, which spreads within the whole plant and leads to a lethal phenotype. However, to date there are no data revealing which viral genes encode for a specific pathogenicity determinant that triggers the plant necrotic response for any torradovirus. In this study we evaluated the influence of three coat protein subunits of ToTV: Vp23, Vp26 and Vp35, transiently expressed from a PVX-based vector, and checked their association with the induction of systemic necrosis in infected *Solanum lycopersicum* L. (*cv. Beta Lux*), a natural host of ToTV.

**Methods:**

To estimate how ToTV coat protein subunits might contribute in plant response to virus infection we over-expressed the proteins from PVX-based vector in tomato and analyzed enzymatic activities related with plant defense response. By doing protein qualitative analysis performed by mass spectrometry we indicated the PR10 in protein fraction with induced ribonuclease activity.

**Results:**

We observed that only the Vp26 enhanced PVX pathogenicity causing severe necrosis of the infected plant. Moreover, we indicated increased RNase and oxidative activities in plants infected with PVX-Vp26 chimeras only. Importantly, we suspected that this increased RNase activity is associated with increased accumulation of PR10 mRNA and products of its translation.

**Conclusions:**

On the basis of the obtained results, we indicated that Vp26 acts as the elicitor of hypersensitive response-like reactions of PVX-Vp26 manifesting with enhanced pathogenicity of the recombined PVX. This might be the first described suspected necrosis determinant of torradoviruses infecting tomatoes.

**Electronic supplementary material:**

The online version of this article (10.1186/s12985-019-1117-9) contains supplementary material, which is available to authorized users.

## Background

Manifestation of virus infection on susceptible plants can be described by the wide range of disease symptoms that develop on the host infected by the pathogenic agent and that lead finally to the lowering of a crop yield and/or its consumer value. Therefore, much effort has been applied to mapping and identifying the host genes involved directly in plant defense responses to virus infection. However, the second component of the interactions—the virus-derived pathogenicity determinants—need to be defined and isolated to identify mechanisms of disease initiation at the very early stages of infection. The well-described RNA silencing suppressor of the cucumber mosaic virus, the 2b protein, which influences local and systemic viral movement, has been reported as a virulence-determining factor in *Nicotiana tabacum*, where it induces mild mosaic symptoms on upper, non-inoculated leaves [1]. In the cited research the 2b protein was described to act as virulence determinant involved in post-transcriptional gene silencing - a well described plant defence mechanism against viruses. Interestingly, it has also been indicated that coat proteins (CPs), in parallel with their structural function, may also play an important role in the elicitation of disease symptoms, for example, one of the biological functions of the p42 protein of melon necrotic spot virus is directly associated with symptom induction in infected melon plants [2].

The hypersensitive response (HR), that in some cases accompanies virus infection, is based generally on the isolation of virus-infected cells from adjacent healthy tissue, thus preventing further spread of such cells within the host [3]. An invading pathogen triggers a cascade of reactions, resulting in a burst of reactive oxygen species (ROS) in infected cells. The level of ROS and strength of the oxygen burst are strictly controlled by cellular peroxidases (POX), superoxide dismutase (SOD) and catalases. The HR was described to be induced by 2b of cucumber mosaic virus interacting with catalase CAT3 in *Arabidopsis thaliana* [4] whereas transient expression of the NSm protein in leaves of Sw-5 tomato or Sw-5 transgenic *Nicotiana benthamiana* resulted in a hypersensitive response [5, 6].

**Fig. 1:**
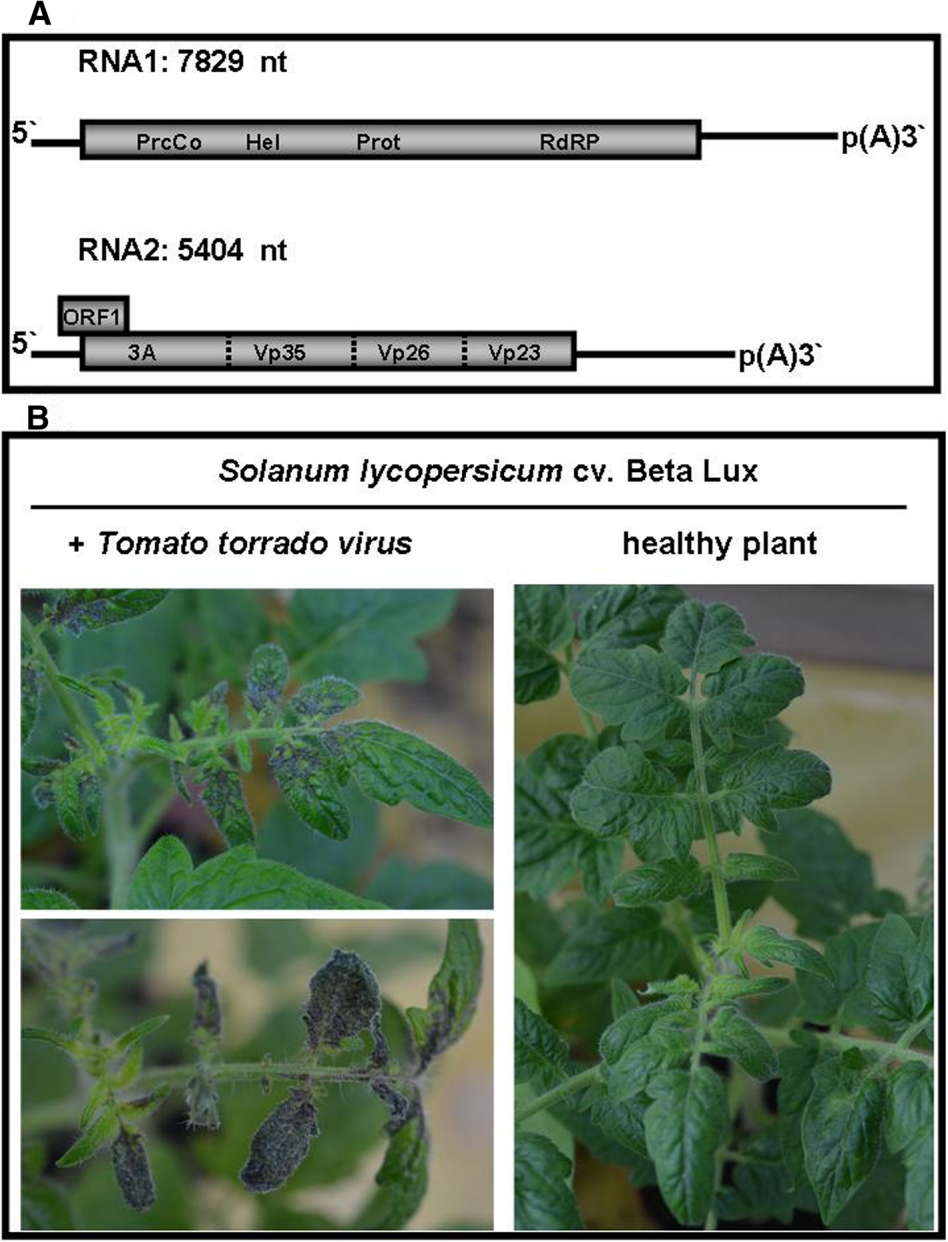
Molecular and biological properties of tomato torrado virus. **a** Schematic representation of the ToTV genome. The genomic material of ToTV comprises two polyadenylated RNA strands, RNA1 and RNA2, composed of 7829 nucleotides and 5404 nucleotides, respectively. RNA1 encodes for protease cofactor (PrcCo), helicase (Hel), viral protease (Pro) and RNA-dependent RNA polymerase (RdRP). RNA2 encodes for ORF1 (necessary for systemic infection) and ORF2 encoding movement protein (3A) followed by three capsid protein (CP) subunits (Vp35, Vp26 and Vp23). Dashed lines indicate borders of each CP subunit. **b** Symptoms induced by ToTV in *Solanum lycopersicum cv. Beta Lux*

*Tomato torrado virus* (ToTV) has been classified within the genus *Torradovirus* in the family *Secoviridae* [7]. Torradoviruses have a bipartite genome consisting of positive sense (+), single-stranded RNAs encapsidated into nonenveloped virions [7]. RNA1 (7829 nucleotides-nt) encodes for a long polyprotein (2158 amino acids) involved in virus replication. RNA2 (5404 nt) encodes for two open reading frames (ORFs): ORF1 (187 amino acids), that is necessary for systemic infection [8] and a long ORF encoding a polyprotein (1198 amino acids) that can be proteolytically processed into a movement protein (3A) and three coat protein (CP) subunits: Vp35, Vp26 and Vp23 (Fig. 1a). In the presence of viral proteinase, both ToTV polyproteins are cleaved to functional proteins; however, the precise cleavage sites have not yet been identified. In a field and greenhouse conditions ToTV is transmitted by insects. Once the pathogen has been acquired by its vector, the whitefly [9–11], it can be transmitted efficiently to a host, particularly *Solanum lycopersicum*. The virus spreads within the tomato plants, leading to severe necrosis of the leaves (Fig. 1b), stems and even fruits [12]. It has been indicated that the virus may infect and accumulate in weed species, which serve as a potential source of infection of economically important plant-hosts of the pathogen [13].

Still little is known about pathogenicity determinants encoded by torradoviruses in a context of disease symptoms induced by the pathogens on a tomato and other host plants. Recently published data concerning ToTV pathogenicity described that a single amino acid mutation in the movement protein domain (the 3A) of the virus can have a substantial impact on virus infectivity in tomato and *N. benthamiana* [14]. In 2016 we described that N-terminal fragment of polyprotein encoded by ToTV RNA1 induces HR-like reaction in *N. benthamiana* [15]. However, studies concerning torradoviruses have focused, in majority, on inter- and intraspecies genetic diversity [16–20] rather than on putative proteins that may be involved in pathogenesis and plant–virus interactions.

In this study, we analyzed the possible role of the ToTV CP subunits Vp23, Vp26 and Vp35 in the induction of systemic necrosis in *S. lycopersicum* (*cv. Beta Lux*). We used a heterologous protein expression system based on a modified potato virus X (PVX)-derived vector to verify the plant response to the ToTV Vps (coat protein subunits). We showed that Vp26 in particular induces necrosis in plants when expressed from the PVX-based vector.

To examine the impact of the Vp26 protein on cellular defense mechanisms (anti-oxidative: POX as well as on RNase activities), we conducted in-gel enzymatic activity assays, and subsequently we analyzed changes in the level of a transcript encoding pathogenesis-related (PR) protein with putative RNase activities, particularly the SlPR10.

## Methods

### Virus isolate and plant material

Virus isolate (ToTV-Wal′03 [10] or ToTV-Kra [17]) was maintained on *Nicotiana benthamiana* and *S. lycopersicum* (*cv. Beta Lux*), grown in greenhouse conditions. When the *S. lycopersicum* (*cv. Beta Lux*) is challenged by ToTV, it develops systemic necrosis. Infectious clones of the virus (p35ToTV-Kra) were used for manipulations of the ToTV Vp26 [21].

### Total RNA extraction and reverse transcription

Total RNA was isolated according to the protocol described previously [22]. The RNA was dissolved in RNase-free water and stored for further use.

For cloning purposes, cDNA was synthesized using 2 μg of total RNA, 1 μl of 50 μM oligo(dT)_18_ and 200 U SuperScript III reverse transcriptase (Thermo Fisher Scientific, Waltham, MA, USA) according to the manufacturer’s protocol.

For gene expression purposes, cDNA was synthesized on 2 μg of the DNA-free RNA in the presence of 200 ng of random primers and 200 U of RevertAid Reverse Transcriptase (Thermo Fisher Scientific) following the manufacturer’s protocol. The obtained mixture was diluted in a 1:1 ratio with nuclease-free water and used for a real-time quantitative PCR.

### Cloning and expression of coat protein subunit cDNAs

Sequences of all primers used in the study are indicated in Additional file 1: Table S1. PCR amplifications of cDNA fragments encoding viral capsid Vp35, Vp26 and Vp23 subunits were performed with three sets of primer pairs—Vp35SmaF/Vp35SmaR, Vp26SmaF/Vp26SmaR and Vp23SmaF/Vp23SmaR—respectively. Each PCR was done using 2.5 U of *PfuUltra* II Fusion DNA polymerase (Agilent Technologies, Santa Clara, CA, USA), primers and 1 μl of the cDNA. The PCR products were digested with FastDigest *SmaI* (Thermo Fisher Scientific) and ligated with *SmaI*-digested and alkaline phosphatase-treated PVX-derived expression vector pgR107 (kindly provided by Prof. David Baulcombe). The ligation mixture was used for the transformation of competent *E. coli* TOP10 cells (Thermo Fisher Scientific). Sequence-verified plasmids were used for the transformation of a competent *A. tumefaciens* GV3101 strain (with the helper pSoup plasmid), grown for 2–3 days at 28 °C on LB-agar plates supplemented with 50 μg/ml of kanamycin and 5 μg/ml of tetracycline. A single bacterial colony was selected using a sterile toothpick and inoculated to a 3-week-old lower leaf (punched six times around a leaf’s main vein) of *S. lycopersicum* (*cv. Beta Lux*) seedlings. Plants were maintained at 28 °C in temperature-controlled greenhouse chambers with a 14-h/10-h light/dark photoperiod. When the infection symptoms became apparent, they were photographed, and the plant material was collected for subsequent analyses. Agro-inoculation was conducted in four replicates with PVX-Vp35, PVX-Vp26 and PVX-Vp23 constructs, whereas plants infected with unmodified PVX vector served as a negative control.

Alternatively, the plants were inoculated by means of agro-infiltration. For this purpose, several *A. tumefaciens* colonies were picked and grown at 28 °C for 24–48 h in liquid LB medium with the aforementioned antibiotics. The bacteria were then harvested and resuspended in infiltrating medium (10 mM MgCl_2_, 0.5 μM acetosyringone, 10 mM MES [2-(N-morpholino)-ethanesulfonic acid], pH 5.8). After 2 h’ incubation at room temperature, the bacteria were brought to an OD_600_ 1.0 and used to infiltrate the *S. lycopersicum* seedlings.

### Gene expression and virus accumulation analyses

Expression of the ToTV CP subunits from PVX-based vector in *S. lycopersicum* (*cv. Beta Lux*) was confirmed by PCR using the primers and thermal cycling conditions mentioned above. Briefly, the reaction mixture contained 1× reaction buffer, 200 μM dNTPs, 500 nM forward and reverse primers, 1 μl (ca. 50 ng) cDNA, and 0.5 U of Allegro *Taq* DNA polymerase (Novazym, Poland). The PCR products were separated on a 1% agarose gel, eluted and sequence verified.

Accumulation of genomic RNA of the PVX-chimera was analyzed by means of a reverse transcription real-time PCR (RT-qPCR) approach with primers PVX1/PVX2 (for quantitation of PVX RNA-dependent RNA polymerase ORF). For this purpose, 1 μl of obtained cDNA was added to 9 μl of RT-qPCR master mix containing iTaq™ universal SYBR® Green supermix (Bio-Rad, Hercules, CA, USA) and 0.5 μM forward and reverse primers. The PCR temperature profile was set as recommended by the manufacturer.

The *PR-10* transcript level in the infected tomatoes was estimated by RT-qPCR using primers SlPR10g/SlPR10h. For normalization, the mRNA of the EF1*α* reference gene (SlEF1*α*) was used [22]. Gene expression was analyzed using GenEx software (MultiD, Sweden).

### Immunological detection

The accumulation of PVX was assessed by means of western blot with antibodies specific to the coat protein of the virus. Briefly, two discs (ca. 5 mm in diameter) were cut out of systemic leaves of plants and were homogenized in a presence of 150 μl 2× Laemlli buffer (Thermo Scientific), boiled for 10 min and centrifuged at 13,000 rpm. The resulted lysate (15 μl) was subjected to 12% SDS-PAGE and fractionated proteins were blotted onto PVDF filter. The filter was blocked in PBS buffer with 0.1 Tween (PBS-T) and 5% non-fat milk, washed three times with PBS-T, incubated with primary antibody against PVX coat protein (1:1,000 dilution; LOEWE,Germany) and subsequently with secondary antibody conjugated with horse radish peroxidase (1:25,000 dilution; Agrisera AB, Sweden). The signal was developed in a presence of ECL Bright substrate (Agrisera AB).

### Enzyme activity analyses

In order to assess RNase and antioxidative enzymatic activities, protein extracts were prepared from plants infiltrated with ToTV, PVX or the PVX chimeras. Up to 200 mg of plant material was taken for this purpose and ground in extraction buffer (1 M Tris-HCl pH 7.5 and 20% glycerol) using a micropestle. The resultant homogenate was clarified by centrifugation (13,000 rpm, 30 min, 4 °C) and the supernatant was taken for subsequent analyses. Protein concentration was estimated using the Bradford method [23]. For determining POX and RNase activities, 50 μg of total protein was taken.

The profile of induced RNases was determined as described by Yen and Green [24]. Briefly, protein extracts were resolved on matrix containing acrylamide/N′,N′-methylene-bis-acrylamide and 2.4 mg/mL torula yeast RNA. The gel was placed in running buffer of 1.4% (*w*/*v*) glycine, 27.5 mM Tris, and 0.1% (w/v) SDS. The SDS was removed from the gel after two subsequent washes with 0.01 M Tris-HCl buffer supplemented with 25% (*v*/v) isopropanol, followed by subsequent incubations in 0.01 M and 0.1 M Tris-HCl. The bands corresponding to RNase activity became visible after gel illumination.

The method described previously [25] was used to estimate changes in POX activities. The resolved native PAGE gel was immersed in 50 mM sodium citrate and incubated in the buffer for 30 min. Next, the gel was washed in 1 mM DAB (3,3′-diaminobenzidine) and 0.03% (*w*/*v*) hydrogen peroxide in a citrate buffer that was made just before use. Peroxidase activity was identified by the presence of dark-brown bands.

### Proteomic analyses: Protein identification and mass spectrometry

The bands containing the protein fractions that showed the RNase activity were excised from the gel and analyzed by liquid chromatography coupled with mass spectrometry. Peptides were separated on a nano-Ultra Performance Liquid Chromatography (UPLC) RP-C18 column (Waters, BEH130 C18 column) using a 45-min linear acetonitrile gradient. The column outlet was directly coupled to an electrospray-ionization (ESI) ion source of an Orbitrap Velos mass spectrometer (Thermo Fisher Scientific), working within a regime of data-dependent MS to MS/MS switch.

Raw data files were pre-processed using Mascot Distiller software version 2.4.2.0, MatrixScience). The obtained peptide masses and fragmentation spectra were matched to an NCBI (National Center for Biotechnology Information) non-redundant database (31,002,772 sequences/10668937692 residues), with a *Viridiplantae* filter (1,473,438 sequences) and to a tomato non-redundant database (34,727 sequences/11956401 residues) using the Mascot search engine (Mascot Daemon v. 2.4.0, Mascot Server v. 2.4.1, MatrixScience).

Protein identification was performed using the Mascot search engine (MatrixScience, Boston, MA, USA), with a probability-based algorithm. An expected value threshold of 0.05 was used for analysis.

### Bioinformatic analyses

Sequence alignment of tomato torrado virus Vp26s was carried out using BioEdit software [26].

## Results

### Vp26 triggers local and systemic necrosis in *S. lycopersicum* infected with PVX-Vp26

**Fig. 2:**
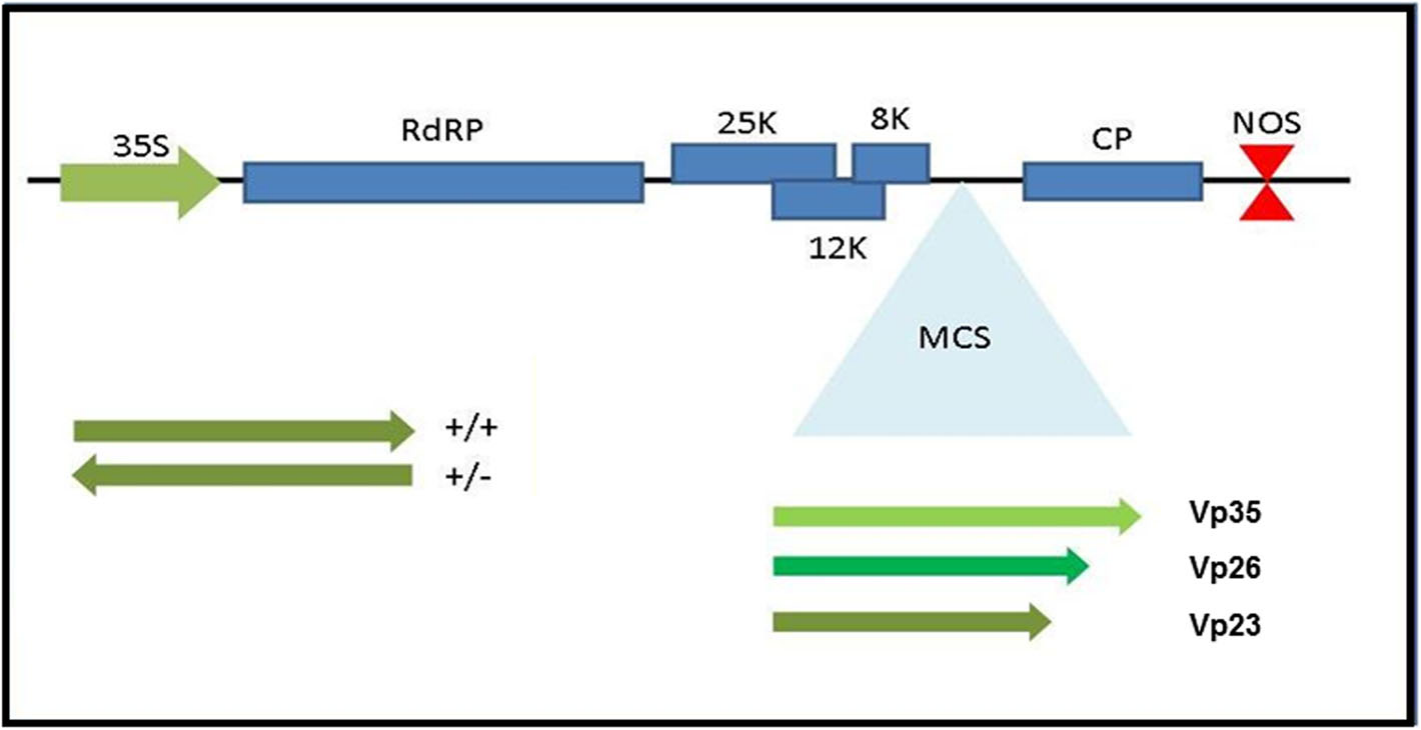
Plasmid constructs used in the study. Five ORFs of potato virus X are indicated (RdRP – replicase, 25K, 12K, 8K – the triple gene block genes, CP – coat protein gene). The multi-cloning site with cloned tomato torrado virus CP ORFs (Vp23, Vp26 and Vp35) is indicated. Orientation of the Vp ORFs is indicated in a sense (+/+) and anti-sense (+/−) orientation

**Fig. 3:**
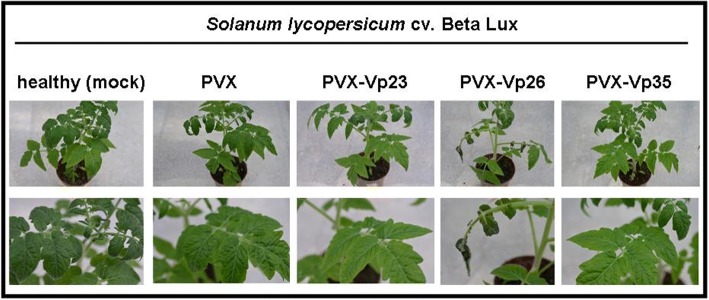
Symptoms manifested in *Solanum lycopersicum* (*cv. Beta Lux*) after infection with potato virus X chimeras. General plant view (upper row) as well as close-ups of systemic leaves (bottom row) are included. Healthy plants (mock) are indicated and compared with plants infected with PVX. The images were recorded at 14 dpi (days post infiltration)

The main goal of the study was to identify ToTV genes that may be involved in the induction of the necrosis on tomato infected with the ToTV (Fig. 1). To express ToTV-derived proteins in tomato, we used a PVX-based expression vector (Fig. 2) [27, 28]. The mentioned PVX does not cause any symptoms of infection in tomato plants (*cv. Beta Lux*) (Fig. 3) and can therefore be used in the studies on the identification of ToTV pathogenicity determinants without risk of inducing nonspecific plant reactions.

The three CP open reading frames (Vp23, Vp26 and V35) from ToTV were introduced into the PVX-based expression vector, creating the following PVX chimeras named hereafter: PVX-Vp23, PVX-Vp26 and PVX-Vp35, respectively. Expression of the inserted transcripts occurred in plants after agro-inoculation of the tomato seedlings with the PVX-Vp constructs. Any PVX infection was monitored visually in systemic leaves on the 6th and 14th day post agro-inoculation; detection of PVX infecting tomato was additionally confirmed by means of RT-PCR.

At six days post inoculation the first necrotic local lesions were visible on the plants infected with PVX-Vp26. The symptoms developed around the inoculation area and gradually migrated along the petiole of the inoculated leaves. Simultaneously, plants inoculated with neither PVX-Vp23 nor PVX-Vp35 displayed any necrotic symptoms within inoculated loci (Table 1).

**Table 1:** Summarized results obtained from PVX-Vp assays in *Solanum lycopersicum* on the 6th and 14th day post agro-inoculation

Symptoms observed on *Solanum lycopersicum* (*cv. Beta Lux*)		PVX-		
		Vp23	Vp26	Vp35
6 dpi	Local and petiole necroses (inoculated leaf)	No	Yes	No
	Systemic leaf necrosis	No	No	No
	Apex necrosis	No	No	No
	Plant stunting	No	No	No
14 dpi	Local and petiole necroses (inoculated leaf)	No	Yes	No
	Systemic leaf necrosis	No	Yes	No
	Apex necrosis	No	Yes	No
	Plant stunting	No	Yes	No

**Fig. 4:**
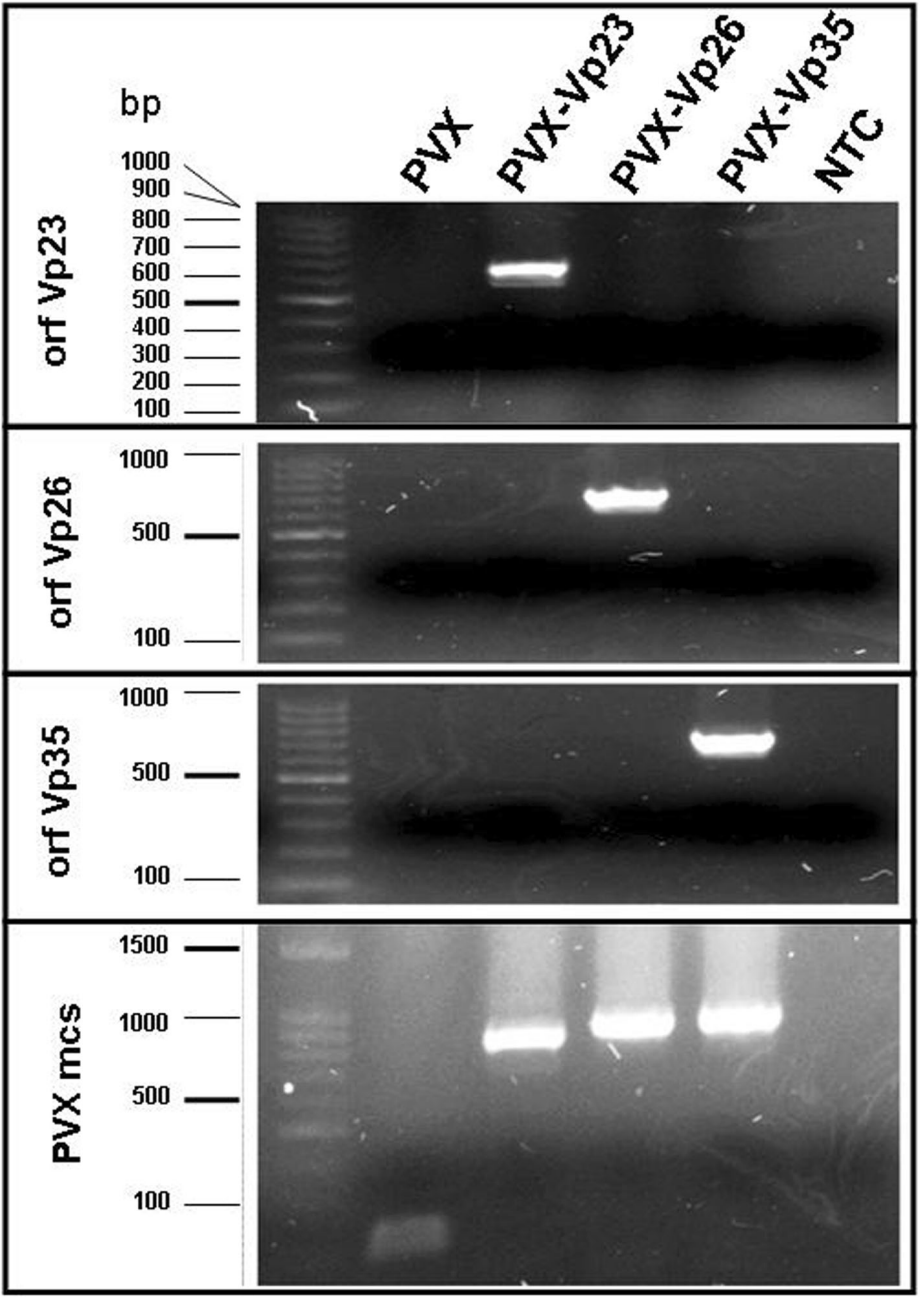
Detection of recombinant potato virus X-Vps in infected tomato plants. Total RNA was extracted from systemic leaves of plants infected with each PVX-Vp individually (14 days post infiltration) and taken for reverse transcriptase polymerase chain reaction (RT-PCR) with corresponding Vp-specific primers. Specific RT-PCR products were detected for Vp23 (650 base pairs, bp), Vp26 (710 bp) and Vp35 (737 bp) in plants infected with PVX-Vp23, PVX-Vp26 and PVX-Vp35, respectively. Primers flanking a multi-cloning site (mcs) of PVX-vector gave a product of ca. 250 bp for the empty vector

The first systemic necrosis developed on the 10th dpi in plants inoculated only with PVX-Vp26; the symptoms became increasingly severe, leading gradually to necrotization of even the lower leaves. Compared with the symptoms induced in plants infected with PVX-Vp26, the plants inoculated with PVX-Vp23 or PVX-Vp35 reacted much less severely when they were examined at the same time points (Fig. 3). To confirm that the Vp23- and Vp35-derived transcripts were expressed from the PVX vector, despite the lack of systemic necrosis in the inoculated plants, a RT-PCR reaction was performed with primers specific for each assessed Vp. The analyses revealed the presence of the Vp23, Vp26 and Vp35 coding sequences in the systemic leaves of plants infected with PVX-Vp23, PVX-Vp26 and PVX-Vp35 (Fig. 4). Importantly, cross-contamination was also excluded. Moreover, western blot with FLAG-specific antibody was done to confirm presence of the FLAG-tagged Vp26 protein in tested plants (see the Additional file 2: Figure S1). To verify that the Vp26-induced necrosis was developed precisely by the properly translated, biologically active Vp26 protein, we inoculated plants with *A. tumefaciens* harboring a PVX-based vector with the Vp26 ORF in an antisense orientation (PVX-Vp26_AS_). This would also provide information as to whether the Vp26-encoding RNA itself served as a necrosis inducer. Subsequent observations of the plants’ response (monitored up to the 30th dpi) revealed extreme necrosis of systemic leaves, stem stunting, and death of the lower leaves of tomato plants infected with PVX-Vp26, but not with PVX-Vp26_AS_. Moreover, a frameshift mutation (adenine residue inserted between positions T314 and A315) introduced into the Vp26 coding sequence (PVX-ΔVp26) resulted in the absence of any systemic necrosis in inoculated plants. Plants expressing Vp23_AS_ and Vp35_AS_ remained symptomless as well, although their open reading frames were detected in PVX- Vp23_AS_ and PVX-Vp35_AS_ (data not shown).

This therefore provides evidence that an RNA-Vp26-derived translation product stimulated the necrotic phenotype in tomato inoculated with PVX-Vp26 and, in the context of the adopted experimental model, is a strong pathogenicity factor.

For subsequent analyses (in-gel activities, gene expression and virus accumulation) plants were collected at 14th dpi.

### Systemic necrosis induced during Vp26 expression is not associated with increased accumulation of PVX RNAs

**Fig. 5:**
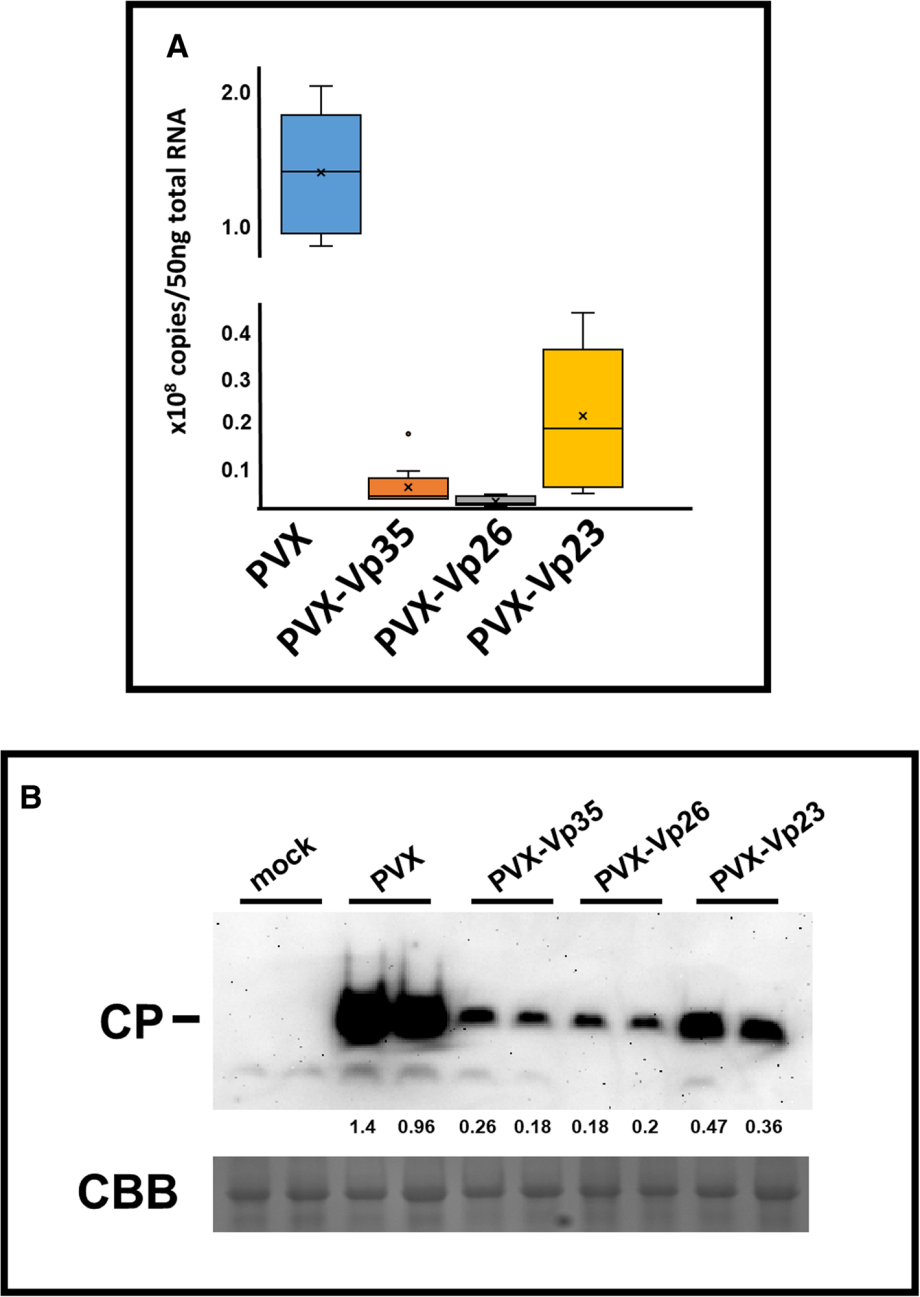
Comparison of the accumulation level of potato virus X (PVX) RNAs in *Solanum lycopersicum* (*cv. Beta Lux*). The analyzed plants were inoculated with PVX-empty construct and PVX chimeras, PVX-Vp23, PVX-Vp26 and PVX-Vp35. **a** Accumulation of PVX genomic RNA (RNA-dependent RNA polymerase - RdRP) was analyzed by menas of quantitative real-time RT-PCR. The RNA used for the analysis was extracted from systemic leaves of tomato seedlings 14 days post infiltration. The copy number of PVX genomic RNA per 50 ng of total RNA was displayed. The mean (the x), the median (the central horizontal bar) and the outliers (the dots) were included in the box charts. **b** Western blot analysis of PVX coat protein (CP) accumulation in *S. lycopersicum* inoculated with tested PVX variants. The normalized densitometry quantitation of chemiluminescence signal was included. The equal protein load was assessed by Coomassie brilliant blue (CBB) staining

To ascertain whether the severity of the enhanced symptoms relating to the PVX-Vp26 chimera was associated with elevated accumulation of the progeny virus, we examined genomic PVX RNA as well as virus coat protein accumulation in the systemic leaves of PVX-infected plants. For this purpose, a total RNA and soluble protein fraction were prepared from the systemic leaves of infiltrated *S. lycopersicum* collected at 14th dpi when the systemic necrosis of systemic leaves were observed. Accumulation of the PVX genomic RNA was measured against the standard curve prepared from serial dilution of plasmid pgR107. In the conducted experiments we observed higher accumulation of unmodified PVX than the PVX chimeras (this was confirmed by assessment of accumulation of additional PVX open reading frames: the 25 K and CP, see the Additional file 3: Figure S2). Moreover, we observed that necrosis-associated PVX-Vp26 accumulated at the level lower than remaining two other PVX variants (Fig. 5a). This was surprising, because we expected that necrosis would result from a high titer of the PVX-Vp26 in tomato. Moreover, the observation was verified again by western blot analysis showing that PVX CP accumulated at the highest level in plants infected with the unmodified PVX (Fig. 5b).

Therefore, it can be concluded that the systemic necrosis induced in a presence of the Vp26 was not caused by higher accumulation of the PVX-Vp26 but resulted rather from Vp26 expression in infected/inoculated plants.

### Vp26 triggers enzymatic activities related to plant defense mechanisms

The POX and RNase activities were compared in plants infected by ToTV, PVX and PVX-Vp26 by means of in-gel activity assays at 7 and 14 dpi.

**Fig. 6:**
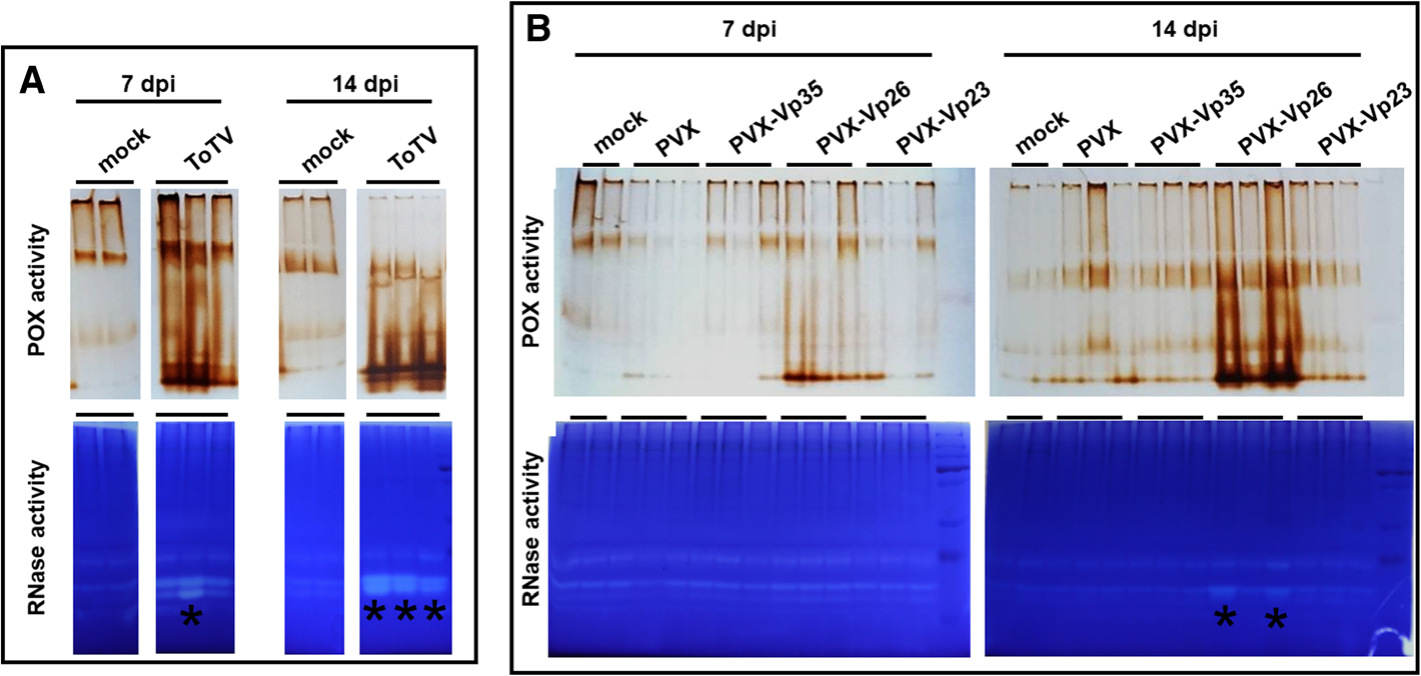
In-gel peroxidase (POX) and ribonuclease (RNase) activity assays. The protein extracts isolated from tomato infected with ToTV (**a**), PVX or PVX chimeras (**b**) at 7 and 14 days post inoculation were resolved under native conditions. Enzymatic activities were indicated after gel staining. The bands of RNase activities taken for MS analysis are marked with the asterisk

Initially we asked if any elevated POX and RNase activities would be observed in tomato infected by ToTV. Indeed, strong POX and RNase activities were detected in plants infected by ToTV already at 7th day after inoculation. At the 14 dpi the activities remained very strong in ToTV-infected plants (Fig. 6a). Next, we checked whether infection of tomato with PVX or PVX-Vp26 would give similar results. Again, the performed experiments indicated that the POX activity increased only in plants infected with PVX-Vp26 (Fig. 6b, the POX panel). At 7 dpi POX activity was observed in protein extracts collected from non-infiltrated and PVX-infected plants, whereas it was evidently higher in plants infected with PVX-Vp26, and at 14 dpi the signal was even stronger (Fig. 6b, the POX panel). Therefore, we concluded that this response was associated with the presence of the Vp26. Increase in POX activity was not observed in plants infected by PVX-Vp35 nor PVX-Vp23.

It was proposed previously that virus replication triggers cellular ribonucleases targeting viral genomic RNA for degradation [29]. Therefore, we assessed whether the expressed Vp26 protein would have an impact on cellular RNase activity as well. First, we checked if ToTV induced stronger RNase activities in infected tomato. In-gel RNase activity assay confirmed that stronger RNase activities were detected in infected tomato at 7 dpi and 14 dpi (Fig. 6a, lower panel). Next, we verified if the increase in RNase activities were also detected in tomatoes infected with the PVX variants. Our results indicated a common band of RNase activity detected in extracts obtained from non-infiltrated, empty PVX- and PVX-Vp-infected plants (Fig. 6b, lower panel). However, at 14 dpi, a band with increased RNase activity was observed in the fractionated protein extracts isolated from plants inoculated with the PVX-Vp26 (Fig. 6b, RNase panel) but not in plants inoculated with other tested PVX recombinants. This induced RNase fraction was excised from the gel and used for subsequent Mass Spectrometry (MS) Orbitrap analyses.

### Vp26 enhances the expression of RNase-like PR-10

The protein fraction characterized by Vp26-induced enhanced RNase activity was subjected to Orbitrap Velos-type mass spectrometry analyses. Table 2 summarizes the results of the Mascot searches. For the purpose of the study, however, proteins with putative or experimentally confirmed RNase activity and PR proteins from the *Solanaceae* family have been subsumed, excluding structural, regulatory or photosynthesis-related records. The MS analysis revealed several protein hits corresponding to PR proteins or PR-like protein families, particularly PR-23 (*S. lycopersicum*), PR-5 (*S. lycopersicum*), p14, PR-10 (*N. tabacum*, *S. virginianum*), P69G (*S. lycopersicum*) and PR-4-like proteins (*S. lycopersicum*). Moreover, the majority of the selected proteins were identified by at least three ions; however, only a single ion has characterized the PR leaf protein 4-like, ribonuclease T2 and protein p14. PR STH-2-like shares the highest amino acid sequence identity with PR-10 proteins from other solanaceous species and was found in the same cluster as them. Therefore, we wondered whether an increase in PR-10 mRNA transcription would accompany a Vp26-induced necrotic response.

**Table 2:** Pathogenesis-related proteins identified after MS/MS analysis

ID	Name	Theoretical MW [Da]	Sequence coverage [%]	Peptides matched	Score rank
gi|19,315	pathogenesis-related protein PR P23 **[Sl]**	**26,024**	40	R.NNCPYTVWAASTPIGGGR.R R.GQTWVINAPR.G R.TNCNFDGAGR.G K.CHAIHCTANINGECPGSLR.V R.VPGGCNNPCTTFGGQQYCCTQGPCGPTDLSR.F R.CPDAYSYPQDDPTSTFTCPSGSTNYR.V	322/441
gi|7,414,370	**pathogenesis-related protein (PR-5 protein)** [Sl]	28,437	25	R.GQSWWFWAPPGTK.M R.TNCNFDGAGR.G R.GWCQTGDCGGVLDCK.G R.CPDAYSYPQDDPTSTFTCQSWTTDYK.I K.CHPIQCTANINGECPGSLR.V	299/334/383
gi|460,404,652	**pathogenesis-related protein STH-2-like** [Sl]	17,472	42	K.GLVLDFDSLVPK.L K.LLSHDVK.S K.SIEIVEGDGGAGSIK.Q K.QMNFVEGGPIK.Y + Oxidation (M) K.IHVIDDK.N K.IHVIDDKNLVTK.Y K.YSLIEGDVLGDK.L K.YSLIEGDVLGDKLESIAYDVK.F K.FEAAGDGGCVCK.T K.GDHVVSEEEHNVGK.G K.AIDLFK.A K.AIEAYLLANPSVYA.-	193/225/599
gi|224,802	**pathogenesis-related, protein p14 [Sl]**	14,589	13	R.AQVGVGPMSWDANLASR.A + Oxidation (M)	119/152/279
gi|372,995,481	pathogenesis-related protein PR-10 [Nt]	17,870	15	K.ALVLDADNLIPK.L K.LHVIDDK.N K.LHVIDDKNLVTK.Y K.YSLIEGDVLGDK.L	45/94/151
gi|4,582,642	ribonuclease T2 [Sl]	27,157	7	R.ESSLDESEFSDLISTMEK.N + Oxidation (M)	128
gi|460,400,304	pathogenesis-related protein P69G [Sl]	80,109	4	K.SSYTVQIASPK.G K.LTYQVTFSK.T R.SPIAVVLALAK.-	110
gi|51,317,936	**pathogenesis-related protein 10** [Sv]	17,690	24	K.QMNFVEGGPIK.Y + Oxidation (M) K.IHVIDDK.N K.YSLIEGDVLGDK.L K.YSLIEGDVLGDKLESINYDIK.F	179
gi|460,402,446	pathogenesis-related leaf protein 4-like [Sl]	18,215	14	R.ASELWVAEKPNYNYGTNQCASGK.V	96

The MS/MS analysis concerns the protein fraction characterized by induced RNase activity. Identified proteins with a score higher than 130 (represented at least twice) are shown in bold. Sl, *Solanum lycopersicum*; Sv, *Solanum virginianum*; Nt*, Nicotiana tabacum*

**Fig. 7:**
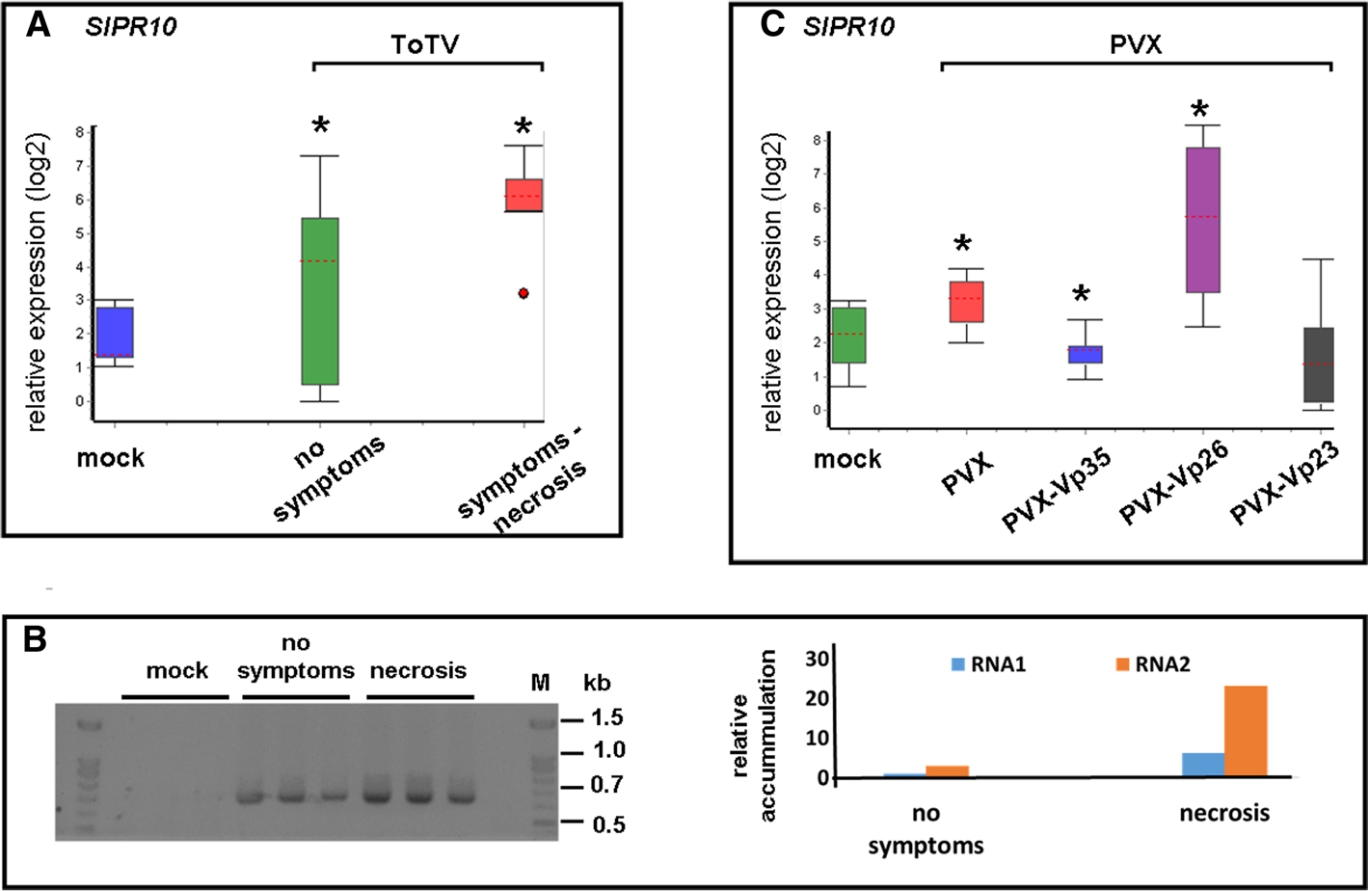
Analysis of expression of the *PR-10* in *Solanum lycopersicum* *cv. Beta Lux* under experimental conditions. **a** Comparison of the relative quantity of the *PR-10* gene in non-inoculated (mock) and ToTV-infected *S. lycopersicum*. Plants expressing no symptoms as well as tomatoes with developed necrosis were included. The red dot describes the outlier. **b** Qualitative (right panel) and quantitative (left panel) detection of ToTV in symptomless and symptomatic *S. lycopersicum*. **c** Fold change of PR-10 mRNA in plants inoculated with the wild-type PVX or with PVX-Vp23, PVX-Vp26 and PVX-Vp35. The expression measurements were normalized against an SlEF1α transcript. Asterisks indicate significant differences as analyzed by the Student’s t-test (*P* < 0.05)

To estimate the level of PR-10 mRNA in plants infected with PVX-Vp26, an RT-qPCR was performed. First, we analyzed the level of PR-10 mRNA in *S. lycopersicum* infected by ToTV. Within the set of ToTV-inoculated tested plants some were found symptomless, whereas at the same time, majority of the plants developed necrosis (observations were done ca. at 10 dpi). In comparison to mock-inoculated plants, we observed increased level of PR-10 mRNA in ToTV-infected *S. lycopersicum* (*cv. Beta Lux*) – both in plants with necrosis and symptomless ones (Fig. 7a; the presence of ToTV in symptomless plants was confirmed by RT-PCR, Fig. 7b). The quantitative analysis confirmed higher accumulation of both RNA1 and RNA2 of ToTV in plants expressing necrosis (Fig. 7b). Therefore the higher expression of the *PR-10* in ToTV-infected plants with developed necrosis might result from higher ToTV accumulation. Next, we checked the expression of the PR-10 in plants infected with the PVX variants.

First, we also asked whether the expression of Vp23 and Vp35 would have any impact at a *PR-10* transcript level in infected plants. The analyses were performed on total RNA isolated from systemic leaves collected at 14 dpi. A priori, however, to exclude the unspecific impact of PVX on the level of the PR-10 mRNA transcript, the expression of the *PR-10* gene was initially estimated by comparing non-infected plants with those infected only with the unmodified PVX. As the level of PR-10 mRNA remained stable in PVX-infected plants (compared with non-inoculated plants), we concluded that infection by the PVX empty vector did not have a significant impact on the expression of *PR-10* (Fig. 7c).

Subsequently, we found that, compared with the plants inoculated with the empty PVX, the relative *PR-10* expression was not influenced by the presence of either PVX-Vp23 or PVX-Vp35 at 14 dpi. However, when the level of the PR-10 transcript was assessed in the plants infected with PVX-Vp26, RT-qPCR analyses revealed an up-regulation of the *PR-10* gene transcription in the infected plants (Fig. 7c). To exclude that the up-regulation of *PR-10* was caused by the Vp26-delivering RNA molecule derived from the PVX backbone, comparative RT-qPCR was conducted on an RNA template isolated from plants infected with PVX-Vp26_AS_. The resulting data indicated similar levels of PR-10 mRNA in plants infected with either PVX-Vp26_AS_ or PVX lacking any insert (data not shown). Therefore, taken together, it can be postulated that *PR-10* expression is up-regulated as a result of the presence of Vp26 protein in infected cells.

## Discussion

In this study, we analyzed the possible role of the three CP subunits of the ToTV capsid in the induction of severe necrotic-reaction characteristics relating to ToTV infection in *S. lycopersicum* (*cv. Beta Lux*). To do so, we initially analyzed whether, and how, the Vps would influence *S. lycopersicum* (*cv. Beta Lux*) when over-expressed from a PVX vector. Exploring further, we wondered which biochemical processes could be induced in a plant in the presence of a selected viral effector.

To achieve the goal we made use of a commonly applied experimental approach based on a PVX-derived expression vector. Generally, the PVX-based model has been used successfully to identify a number of putative disease determinants of RNA viruses [28, 30–33], DNA viruses [34–36] and satellite nucleic acids accompanying helper viruses [37]. However, it should be kept in mind that the PVX-based pathosystem raises the possibility of the occurrence in *N. benthamiana* of unspecific interactions between PVX-derived factors and the protein expressed from the vector [38].

Torrado disease manifests with severe necrosis induced upon infection of *S. lycopersicum* (*cv. Beta Lux*) by ToTV (Fig. 1). The first symptoms of infection are visible at 7–10 dpi and gradually develop to a burn-like phenotype (severe necrosis). As a starting point, we assumed that, as the natural way for ToTV transmission in the environment is via whiteflies, hence fully assembled ToTV virion-particles delivered from the vector’s stylet may be exposed to the cellular environment at the first outset of virus invasion. Moreover, supported by previous reports that CPs can be responsible for the induction of symptoms, or their modulation [39–41], we focused on the influence of three ToTV CP subunits—namely, Vp23, Vp26 and Vp35—on the plant response to these molecules. Indeed, we have shown that the Vp26 subunit of a ToTV CP is responsible for the development of systemic necrosis similar to torrado disease symptoms when the protein was over-expressed heterologously from a PVX-based vector in tomato plants. In contrast to plants infected with ToTV or PVX-Vp26, the PVX, PVX-Vp23 and PVX-Vp35 did not induce any necrosis on the inoculated plants (Fig. 2). It has also been shown that a Vp26-induced necrotic response in tomato was not accompanied by a substantially increased accumulation of PVX-Vp26 RNAs. Experimental data described previously [42] showed that a small 4.8 kDa product of ORF6 of TMV did not enhance either TMV or engineered PVX-ORF6 RNA accumulation in *N. benthamiana* at 12 dpi, although the protein induced severe systemic necrosis in this host. Cao et al. [30] described that the Pns10 protein of rice dwarf virus enhanced the pathogenicity of chimeric PVX without increasing its accumulation. However, Aguilar et al. [38] highlighted the substantial increase in accumulation of the subgenomic RNA of PVX (particularly CP ORF) expressing heterologous viral suppressors of RNA silencing in *N. benthamiana*.

Next, we analyzed the impact of Vp26 on POX cellular activities. In a classic model, however, the HR limits the occurrence of a pathogen to several cells, restricting virus movement by inducing programmed cell death around the site of infection. The mechanisms are related to, among others, the production of ROS [3, 43]. We have shown that at 14 dpi a strong POX activity was accompanied by the infection of ToTV. Moreover, the strong POX activity was observed in plants expressing the Vp26 protein from PVX backbone compared with plants infected with an empty PVX vector. The observations align with data presented by Dieng [44], where the antioxidant activity of POX was measured in tomato infected with TYLCV (tomato yellow leaf curl virus). The same was observed by Clarke et al. [45], who described an immediate increase in POX activity in *Phaseolus vulgaris* upon infection by white clover mosaic potexvirus.

We also observed that protein extracts isolated from plants inoculated with ToTV or PVX-Vp26 showed a significant induction of RNase activities compared with plants inoculated with an empty PVX vector. The T2 ribonuclease was the only classic RNase from *S. lycopersicum* identified in the samples (Table 2). The enzymes belonging to the T2 RNase family are divided into two subfamilies (S-RNases and S-like RNases) [46] and, as was proposed for *Arabidopsis thaliana*, the T2 RNase is essential for a housekeeping function during ribosomal RNA recycling [47]. Moreover, 23-kDa RNS1, a member of the T2 RNases, was induced after wounding in *A. thaliana*, both locally and systemically [48]. As all the mentioned plants used in our study were inoculated mechanically after introducing *A. tumefaciens* into the leaves, this may have affected the T2 activity in the experimental tomato plants. Therefore, we omitted the enzyme in our further analyses.

In the induced RNAse fraction by Vp26, several PR proteins were identified. MS analysis pointed additionally to the pathogenesis protein PR10a (formerly STH-2), which was chosen for further transcript profiling. Previously, it was shown that *PR10* expression from *Capsicum annuum* had been induced during the incompatible interaction with TMV [49] in pepper as well as in *Solanum virginianum* (*S. sutattense*), where the SsPR10 was stress- and pathogen-induced [50]. Vp26 also triggered an increase in a PR STH-2-like transcript in *S. lycopersicum*, and thus may function as the elicitor of the STH-2-like response. Notably, Choi et al. [51] previously reported that the RNase-activity possessing PR-10 was essential to triggering an HR response, and the silencing of its expression compromised resistance in pepper. This correlation between induction of HR by RNases was also described by Kim and Hwang [52] for pathogenesis-related protein 4c isolated from pepper. The PR4c (possessing ribonuclease activity) caused HR cell death in pepper leaves, which was accompanied by enhanced accumulation of H_2_O_2_ and significant induction of some defense-response genes. This aligns with the data presented here in our study.

## Conclusions

In summary, to our knowledge, this is the first report describing the molecular fundamentals of torrado disease pathogenesis regarding the CPs of the ToTV. Using the experimental system based on a PVX-Vp26 recombinant virus, we showed that Vp26 may be considered as an effector protein inducing a lethal systemic necrosis phenotype in plants where the molecule is expressed in a context of virus infection.

## Additional files

Additional file 1: **Table S1.** Sequences of the primers used in the study. (DOC 42 kb)

Additional file 2: **Figure S1.** Expression of the FLAG-tagged Vp26 protein from pgR107 vector in *Solanum lycopersicum* (*cv. Beta Lux*) and *Nicotiana benthamiana*. (a) Schematic presentation of the used Vp26 variants (green bar represents Vp26 protein; red boxes indicate the FLAG tagging sequence) expressed in tomato and tobacco. Modified Vp26 induced local HR in *S. lycopersicum* and strong necrotic systemic lesions in infected leaves of *N. benthamiana*. (b) Western blot of FLAG-tag fused Vp26 proteins extracted from systemic leaves of tomato (Sl1 and Sl2) and tobacco (Nb). The engineered Vp26 variants were identified using anti-FLAG M2 monoclonal antibodies in two randomly selected *S. lycopersicum* and *N. benthamiana* plants expressing Vp26 with FLAG fused to its N-terminus. (c) RNA identification of Vp26 FLAG-tagged variants in systemic leaves of infected tomato (Sl1 and Sl2) and tobacco (Nb). A one-step RT-PCR reaction was performed with primers specific for the Vp26 coding sequence, and the RNA template extracted from the systemic leaves of *S. lycopersicum* and *N. benthamiana* infected with the pgR107 vector harboring the Vp26 FLAG-tagged variants. The asterisk indicates the Vp26-specific amplification product. (TIF 497 kb)

Additional file 3: **Figure S2.** Comparison of the accumulation level of potato virus X (PVX) RNAs in *Solanum lycopersicum* (*cv. Beta Lux*). The analyzed plants were inoculated with PVX-empty construct and PVX chimeras, PVX-Vp23, PVX-Vp26 and PVX-Vp35. Accumulation of three PVX open reading frames coding RNA-dependent RNA polymerase (RdRP), 25 K protein and coat protein (CP) were analyzed. The RNA used for the analysis was extracted from systemic leaves of tomato seedlings at 14 days post infiltration (dpi). The copy number of PVX genomic RNA per 50 ng of total RNA was displayed. The mean (the x), the median (the central horizontal bar) and the outliers (the dots) were included in the box charts. (TIF 74 kb)

## Abbreviations

CP: Coat protein
DAB: 3,3′-diaminobenzidine
dpi: Days post inoculation/infiltration
HR: Hypersensitive response
MS: Mass spectrometry
NBT: Nitro blue tetrazolium chloride
NCBI: National Center for Biotechnology Information
ORF: Open reading frame
POX: Peroxidase
PR: Pathogenesis-related protein
PVX: Potato virus X
RdRP: RNA-dependent RNA polymerase
RNase: Ribonuclease
ROS: Reactive oxygen species
RT-PCR: Reverse transcription PCR
RT-qPCR: Reverse transcription real-time PCR
SDS-PAGE: Sodium dodecyl sulfate-polyacrylamide gel electrophoresis
SOD: Superoxide dismutase
TEMED: N,N,N′,N′-tetramethylethylenediamine
TMV: Tobbacco mosaic virus
ToTV: Tomato torrado virus
TYLCV: Tomato yellow leaf curl virus
