# Supplementary material for: Contribution of Tomato torrado virus Vp26 coat protein subunit to systemic necrosis induction and virus infectivity in Solanum lycopersicum
Source: Virol J. 2019 Jan 14;16:9. doi: 10.1186/s12985-019-1117-9 (PMC6332883; doi:10.1186/s12985-019-1117-9)
Supplement: Supplementary file 1 — Table S1. Sequences of the primers used in the study. (DOC 42 kb) [file 12985_2019_1117_MOESM1_ESM.doc]

| **#** | **PRIMER ID** | **SEQUENCE 5`🡪3`** | **Notes** |
| --- | --- | --- | --- |
| **OVER-EXPRESSION OF CP SUBUNITS** | | | |
| 1 | VP35SmaF | GTGCCCGGG*ATG*GTGGCCCAAACTAGTGTG | Start codon is in italic, *SmaI* restriction site is underlined, stop codon is in bold. |
| 2 | VP35SmaR | ATGCCCGGG**CTA**ACGAGGAGGCTGCATTG3 |
| 3 | VP26SmaF | AACCCCGGG*ATG*GCACAATTTGGTATGAAC3 |
| 4 | VP26SmaR | ATGCCCGGG**CTA**CTTTCCTTTCTCATCAAATG |
| 5 | VP23SmaF | TACCCCGGG*ATG*TTTTCATATGGGGCTGTAC |
| 6 | VP23SmaR | TAGCCCGGG**CTA**ATTTTCAAAACTCCTTAG |
| 7 | pgrF | CAATCACAGTGTTGGCTTGC | Sequencing an insert in pgR107 vector |
| 8 | pgrR | GACCCTATGGGCTGTGTTG |
| **QUANTITATIVE REAL-TIME RT-PCR** | | | |
| 9 | PVX1 | GGATAGGAGTGGAACAATGA | for quantitation of PVX RNA-dependent RNA polymerase ORF |
| 10 | PVX2 | CAATTTCTCTCAATGCCTTC |
| 11 | SlPR10g | CAACCACAATTGCCCCAACA | Expression of SlPR10 in tomato |
| 12 | SlPR10h | TCTCCCTCAACAATCTCAATGC |
| **MUTAGENESIS** | | | |
| 13 | ToFlagInsF | GGACTGGGTACACTGAACACAGATTACAAGGATGACGACGATAAGAGCATGTCCAC | Insertion FLAG coding sequence |
| 14 | ToFlagInsR | GTGGACATGCTCTTATCGTCGTCATCCTTGTAATCTGTGTTCAGTGTACCCAGTCC |
| 15 | Vp26_1ntS | GTCCACCCCTCATGACATTTTAAAATATGCCGCATGTTATTTG | Frame shift insertion within Vp26 |
| 26 | Vp26_1ntAS | CAAATAACATGCGGCATATTTTAAAATGTCATGAGG GGTGGAC |

**Table ST1. Sequences of the primers used in the study.**
