# Supplementary figures and images for: Contribution of Tomato torrado virus Vp26 coat protein subunit to systemic necrosis induction and virus infectivity in Solanum lycopersicum
Source: Virol J. 2019 Jan 14;16:9. doi: 10.1186/s12985-019-1117-9 (PMC6332883; doi:10.1186/s12985-019-1117-9)

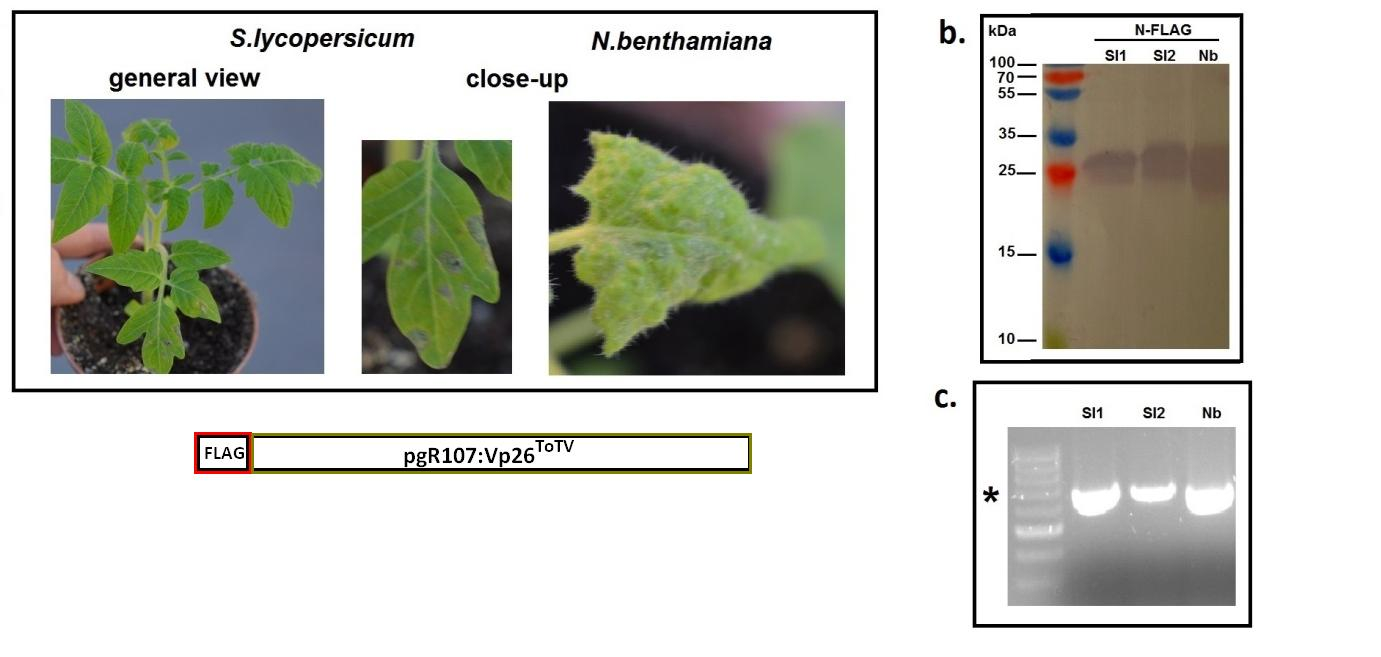

Supplement: Supplementary file 2 — Figure S1. Expression of the FLAG-tagged Vp26 protein from pgR107 vector in Solanum lycopersicum (cv. Beta Lux) and Nicotiana benthamiana. (a) Schematic presentation of the used Vp26 variants (green bar represents Vp26 protein; red boxes indicate the FLAG tagging sequence) expressed in tomato and tobacco. Modified Vp26 induced local HR in S. lycopersicum and strong necrotic systemic lesions in infected leaves of N. benthamiana. (b) Western blot of FLAG-tag fused Vp26 proteins extracted from systemic leaves of tomato (Sl1 and Sl2) and tobacco (Nb). The engineered Vp26 variants were identified using anti-FLAG M2 monoclonal antibodies in two randomly selected S. lycopersicum and N. benthamiana plants expressing Vp26 with FLAG fused to its N-terminus. (c) RNA identification of Vp26 FLAG-tagged variants in systemic leaves of infected tomato (Sl1 and Sl2) and tobacco (Nb). A one-step RT-PCR reaction was performed with primers specific for the Vp26 coding sequence, and the RNA template extracted from the systemic leaves of S. lycopersicum and N. benthamiana infected with the pgR107 vector harboring the Vp26 FLAG-tagged variants. The asterisk indicates the Vp26-specific amplification product. (TIF 497 kb) [file 12985_2019_1117_MOESM2_ESM.tif]

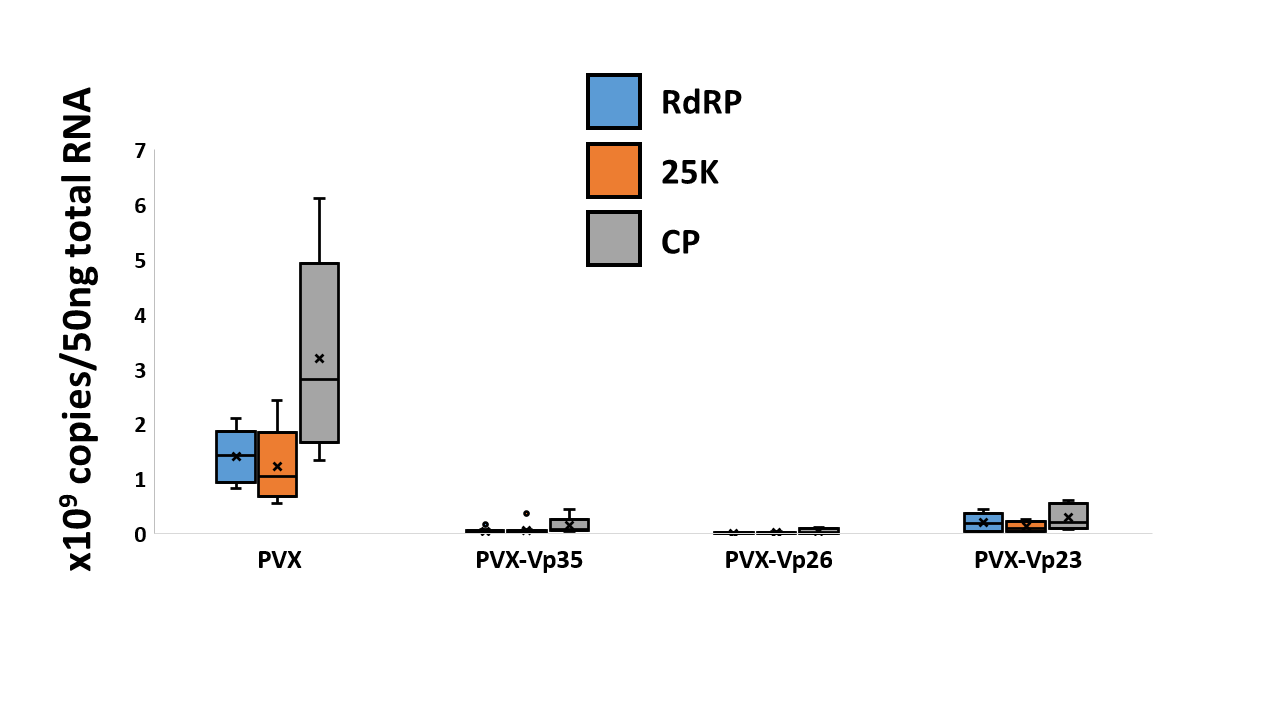

Supplement: Supplementary file 3 — Figure S2. Comparison of the accumulation level of potato virus X (PVX) RNAs in Solanum lycopersicum (cv. Beta Lux). The analyzed plants were inoculated with PVX-empty construct and PVX chimeras, PVX-Vp23, PVX-Vp26 and PVX-Vp35. Accumulation of three PVX open reading frames coding RNA-dependent RNA polymerase (RdRP), 25 K protein and coat protein (CP) were analyzed. The RNA used for the analysis was extracted from systemic leaves of tomato seedlings at 14 days post infiltration (dpi). The copy number of PVX genomic RNA per 50 ng of total RNA was displayed. The mean (the x), the median (the central horizontal bar) and the outliers (the dots) were included in the box charts. (TIF 74 kb) [file 12985_2019_1117_MOESM3_ESM.tif]
